# *Lactobacillus rhamnosus* could inhibit *Porphyromonas gingivalis* derived CXCL8 attenuation

**DOI:** 10.1590/1678-775720150145

**Published:** 2016

**Authors:** Ayşegül Mendi, Sevil Köse, Duygu Uçkan, Gülçin Akca, Derviş Yilmaz, Levent Aral, Sibel Elif Gültekin, Tamer Eroğlu, Emine Kiliç, Sina Uçkan

**Affiliations:** 1 - Gazi University, Faculty of Dentistry, Department of Medical Microbiology, Ankara, Turkey.; 2 - Hacettepe University, PEDI-STEM Center for Stem Cell Research and Development, Ankara, Turkey.; 3 - Gazi University, Faculty of Dentistry, Department of Oral and Maxilofacial Surgery, Ankara, Turkey.; 4 - Gazi University, Faculty of Dentistry, Department of Oral Pathology, Ankara, Turkey.; 5 - Başkent University, Faculty of Dentistry, Department of Oral and Maxillofacial Surgery, Ankara, Turkey.; 6 - Technopolis of Hacettepe University, Hemosoft IT & Training Services, Department of Life Sciences, Ankara, Turkey.

**Keywords:** CXCL8 chemokine, Stroma, Periodontitis, Probiotics, Receptor

## Abstract

An increasing body of evidence suggests that the use of probiotic bacteria is a promising intervention approach for the treatment of inflammatory diseases with a polymicrobial etiology. *P. gingivalis* has been noted to have a different way of interacting with the innate immune response of the host compared to other pathogenic bacteria, which is a recognized feature that inhibits CXCL8 expression.

**Objective:**

The aim of the study was to determine if *P. gingivalis* infection modulates the inflammatory response of gingival stromal stem cells (G-MSSCs), including the release of CXCL8, and the expression of TLRs and if immunomodulatory *L. rhamnosus* ATCC9595 could prevent CXCL8 inhibition in experimental inflammation.

**Material and Methods:**

G-MSSCs were pretreated with *L. rhamnosus* ATCC9595 and then stimulated with *P. gingivalis* ATCC33277. CXCL8 and IL-10 levels were investigated with ELISA and the TLR-4 and 2 were determined through flow cytometer analysis.

**Results:**

CXCL8 was suppressed by *P. gingivalis* and *L. rhamnosus* ATCC9595, whereas incubation with both strains did not abolish CXCL8. *L. rhamnosus* ATCC9595 scaled down the expression of TLR4 and induced TLR2 expression when exposed to *P. gingivalis* stimulation (p<0.01).

**Conclusions:**

These findings provide evidence that *L. rhamnosus* ATCC9595 can modulate the inflammatory signals and could introduce *P. gingivalis* to immune systems by inducing CXCL8 secretion.

## INTRODUCTION

The gram-negative, anaerobic bacterium *Porphyromonas gingivalis* is considered to be one of the key pathogens in periodontitis^20^ ^,^ ^24^. *P. gingivalis* possesses a number of pathogenic properties that enhance growth and survival such as fimbriae, lipopoylsaccharides, and gingipains^24^. Accumulating data shows that gingipains are involved in the regulation of host inflammatory responses. *P. gingivalis* stimulates an innate immune response and induces the expression of inflammatory mediators, but it can downregulate the host immune response at the same time. In other words, *P. gingivalis* has evolved various mechanisms to escape host immune systems by invading host cells and disrupting signaling pathways through cytokine and receptor degrading^18^.

Periodontitis is a common, chronic inflammatory disease resulting from a complex polymicrobial infection in which the disruption of the homeostasis between the subgingival microbiota and the host defense leads to the destruction of the tooth-supporting tissue^25^. As a result of bacterial encounters, the host cells synthesize and release cytokines and chemokines, attracting inflammatory cells to the site of infection^19^ ^,^ ^24^. CXCL8 is an important chemokine that attracts neutrophils to the site of infection. The CXCL8 chemokine is expressed and produced by different cell types such as fibroblasts, neutrophils, endothelial cells, keratinocytes, epithelial cells, and lymphocytes^10^. *P. gingivalis* has a different way of interacting with the host’s innate immune response, compared to other pathogenic, gram-negative bacteria, which is recognized as inhibiting CXCL8 expression. The attenuation of CXCL8 may delay the defense mechanisms of the host and allow *P. gingivalis* to escape the immune system, thus creating more damage to the surrounding tissue^17^.

The ability of the immune system of the host to sense, recognize, and respond to periodontal associated pathogens is an important determinant in the pathogenesis of periodontitis. This ability is largely mediated by the innate immune system via the expression of toll-like receptors (TLRs)^16^. Human gingival fibroblasts express functional TLR2, 3, 4, and 5, and ligands binding to these receptors leads to the secretion of CXCL8^21^. Moreover, TLR2, which recognizes gram-positive bacterial cell walls, is specifically involved in the recognition of *P. gingivalis* ^12^. Studies have shown that *P. gingivalis* could signal via TLR2, TLR4, or both^29^.

Conventional periodontal treatment is often not sufficient by itself to control destructive inflammation, and many patients develop recurrent diseases^1^. This requires the development of novel and effective therapeutic strategies that are adjunctive to clinical periodontal treatment. The use of probiotics is one of the several approaches being considered for the treatment of periodontitis^3^. Probiotic therapy has recently gained massive interest worldwide, due to its potentially beneficial effects on general and oral health as well as for being an important complement to antibiotic treatment. Furthermore, the administration is simple, inexpensive, and safe^23^. Animal and human studies have shown that the use of probiotics is emerging as a potential adjunctive therapy for periodontitis, although the underlying mechanisms remain poorly defined^22^. Our team has supported conducting *in vitro* studies before using probiotics in clinical trials. In this study, the hypothesis tested was that CXCL8 suppression by *P. gingivalis* could be prevented by the probiotic strain *L. rhamnosus* ATCC 9595 through co-aggregation, competitive adhesion, and the expression of TLRs.

## MATERIAL AND METHODS

### Isolation and identification of gingival mesenchymal stromal stem cells

Gingiva samples were obtained from four healthy subjects aged 21 to 24 years old during wisdom-tooth extraction. The experimental protocol was reviewed and approved by the Institutional Ethical Board, and informed consent form was obtained from each subject. Gingival mesenchymal stromal stem cells (G-MSSCs) were isolated and identified according to Zhang, et al.^30^ (2009). Differentiation protocol was conducted according to the protocol described as Pittenger, et al.^26^ (1999).

### Bacteria and growth conditions

*L. rhamnosus* ATCC9595, the probiotic strain, and *P. gingivalis* ATCC33277 were obtained from American Type Culture Collection. The probiotic strain was cultured in MRS broth (de Man, Rogosa, Sharpe, MERCK) under aerobic conditions at 37°C for 18 h. *P. gingivalis* was cultured under anaerobic conditions (80% N_2_, and 10% H_2_) at 37°C in an anaerobic chamber (Electotek Anaerobic workstation, UK) and maintained on Schaedlar Agar supplemented with hemin (5 µg mL^–1^), 5% defibrinated sheep blood, and menadione (1 µg ml^–1^). The number of bacteria at the beginning of the experiment was adjusted to McFarland 2 (~1×10^8^) and expressed as cfu mL^–1^ by the serial dilution technique.

### Auto- and co-aggregation assays

The auto-aggregation assay was performed according to the methods developed by Del Re, et al.^9^ (2000) with certain modifications. Briefly, bacteria were grown for 18 h at 37°C with the appropriate growth medium. The culture was harvested by centrifugation at 5000 g for 15 min, washed twice, and resuspended in phosphate-buffered saline (PBS) to give viable counts of approximately 10^8^ cfu mL^-1^ . Cell suspensions (4 mL) were mixed by vortexing for 10 s, and auto-aggregation was determined during 4 h of incubation at room temperature. At the end of the incubation period, 0.1 mL of the upper suspension was transferred to another tube with 0.9 mL of PBS, and the absorbance (A) was measured at 600 nm. The auto-aggregation percentage was expressed as (1–(A_t_/A_0_))×100, where A_t_ represents the absorbance at the end of incubation time and A_0_ the absorbance at t=0.

The method for preparing cell suspensons for co-aggregation was the same as that for the auto-aggregation assay. Equal volumes (2 mL) of each cell suspension were mixed together in pairs by vortexing for 10 s. Control tubes containing 4 mL of each bacterial suspension on its own were set up at the same time. The absorbance (A) of the suspensions at 600 nm was measured after mixing and after 4 h of incubation at room temperature. Samples were taken in the same way as in the auto-aggregation assay. The percentage of co-aggregation was calculated using the equation given by Handley, et al.^15^ (1987):

Coaggregation (%)=((Ax+Ay)/2−A(x+y))/((Ax+Ay)/2), where x and y represent each of the two strains in the control tubes and (x+y) represents the mixture.

### Competitive adhesion of bacterial strains to G-MSSCs

For the competitive adherence assay, a total cell number of 200 000 G-MSSCs per well were seeded on glass coverslips and incubated for 24 h in 6 well plates. Before the experiment, the culture medium was changed with antibiotic-free medium. G-MSSCs were challenged with bacteria at a multiplicity of infection (MOI) of 1:100 (10^8^ bacteria well^-1^) at 37°C in 5% CO_2_ for 1 h. *L. rhamnosus* and *P. gingivalis* were provided equal chances to bind at the same ratio and at the same time^8^. At the end of 1 h, the incubation monolayers were fixed and prepared for light microscopy, as described by Bernet, et al.^2^ (1993). For each monolayer on a glass coverslip, the numbers of adherent bacteria on 100 different, randomly selected cells were evaluated.

### Effect of *L. rhamnosus* on G-MSSCs upon IFN-γ or *P. gingivalis* stimulation

G-MSSCs were allowed to attach to and grow on 24-well tissue culture plates (50 000 cells well^-1^) containing culture medium (Costar, Corning, USA). After 24 h of incubation, the medium was discarded, washed with PBS, and replaced with new medium without Pen/Strep. cells that were pretreated with *L. rhamnosus* (MOI 1:100) for 12 h at 37°C and 5% CO_2_. Subsequently, the culture wells were washed with PBS and then stimulated with 20 ng mL-1 IFN-γ (Invitrogen, USA) or *P. gingivalis* (MOI: 1:100) for another 12 h. At the end of the experiment, culture supernatants were harvested to determine their cytokine levels, and the cells were prepared for detection of TLR expression by flow cytometer.

### Determination of cytokine production

The co-culture supernatants were collected and centrifuged at 4000 g for 3 min at 4°C. The amounts of CXCL8 and IL-10 secreted into the medium during the co-culturing with the bacteria were measured by using the Human Ultrasensitive ELISA Kit (Invitrogen, USA). The culture supernatants were diluted according to the manufacturer’s instructions. The ELISA limits were 0–25 pg mL^-1^ for CXCL8 and 0–50 pg mL^-1^ for IL-10.

### TLR-4 and TLR-2 analysis by flow cytometer

G-MSSCs were prepared by treatment with Trypsin/EDTA (Invitrogen, USA), transferred to fluorescence-activated cell sorter (FACS) tubes, centrifuged (200 g × 5 min), and washed with PBS. The cells were fixed by adding 2 mL of FACS lysing solution (BD Bioscience, Heidelberg, Germany), then briefly vortexed and incubated for 10 min at room temperature in dark. G-MSSCs were centrifuged (200 g × 5 min) and washed with 2 mL of wash buffer (1% BSA, 0.1 % NaN_3_ PBS). Then, the cells were stained for expression of TLR-2 and TLR-4 on G-MSSCs with anti-TLR-2 FITC and anti-TLR-4 PE antibodies (eBioscience, USA). The harvested cells were incubated within the specific antibody at 4°C for 30 min and labeled with isotype IgG, after which they were used as controls. Appropriate isotype controls (IgG-2a) were also used in each case. Positively stained cells were counted according to their fluorescence and light-scattering properties on a FACS Aria flow cytometer system (CEllQuest ProSoftware, BD Biosciences, Heidelberg, Germany). The experiments were done in triplicate; representative histograms are presented in the Results section.

### Data analysis

Aggregation, adhesion, ELISA, and flow cytometer assays were carried out in two individual experiments involving 3 replicates. Auto- and co-aggregation was analyzed by Spearman’s rho test, whereas Mann-Whitney U test was applied for comparing adhesion and competitive adhesion assays. CXCL8 and IL-10 determination from culture supernatants were analyzed by comparing the G-MSSCs with bacteria treated cells and IFN induced groups using Mann-Whitney U and Kruskal-Wallis test. TLR expression on cell surfaces by IFN-g and/or *P. gingivalis* ATCC33277 and ability of the probiotic strain to reduce TLR-4 expression were investigated by comparing two proportions by Z-test. A value of p<0.01 and p<0.05 was considered to be statistically verified.

## RESULTS

### Gingiva-derived G-MSSCs identified as MSCs

**Figure 1:**
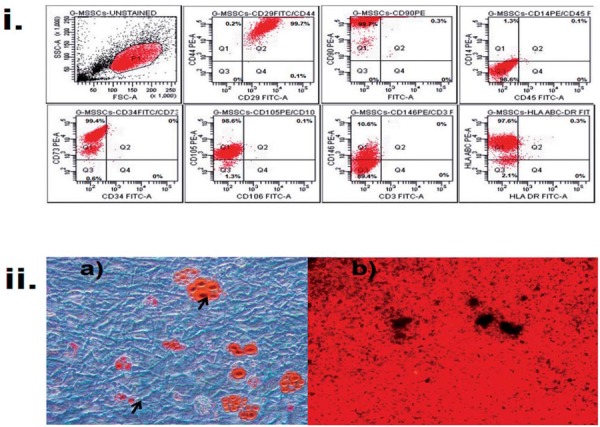
Gingival stromal stem cells (G-MSSCs) showed mesenchymal stem cell properties. i) CD44, CD29, CD106, CD105, CD146, and CD90 are mesenchymal stem cell surface receptors that were detected on G-MSSCs, except CD106. CD106 is a subpopulation of mesenchymal stem cells that addresses immunomodulatory properties. Hematopoietic stem cell surface receptors CD14, CD34, CD45; CD3, HLA DR were found to be negative. Expression of CD73 as a cluster of differentiation and HLA-ABC as an evidence of inflammatory condition was found to be positive ii) G-MSSCs have low adipogenic differentiation potential, whereas osteogenic differentiation was strong (a) Adipogenic differentiation: The black arrows show positively stained G-MSSCs for lipid vesicules with Oil Red O stain. (b) Osteogenic differentiation: The calcium granules were stained black with Alizarin Red stain in the osteogenic differentiation medium on the G-MSSCs (40x, Olympus CKX41, Tokyo, Japan)

The expansion of G-MSSCs in culture was successful for at least eight passages in all of the samples. The common MSC markers CD106, CD105, CD73, CD29, CD90, CD146, and CD44 and the hematopoietic markers, CD3, CD45, CD14, HLADR, HLA-ABC, and CD34, were tested, suggesting a mesenchymal origin for the cells (Figure 1-i).

### G-MSSCs show adipogenic and osteogenic differentiation capacity, but potency is low

Around day 10 (range of 8–14) of exposure to adipogenic medium, very small lipid droplets were barely visible by inverted microscope within the bulk of the cells obtained from gingival tissues (Figure 1-ii.a). Oil Red O staining was performed on the culture in adipogenic medium on day 30. The morphology and staining characteristics of the differentiating cells were consistent with a preadipocyte phenotype. Small, dark deposits were visible on the culture plates of G-MSSCs after 7–10 days, and they increased in the following days within osteogenic differentiation culture. Amorphous deposits were present on the plates by day 30. Calcium mineralization was confirmed by positive Alizarin Red S staining at day 30 of culture, indicating the *in vitro* osteogenic potential of the cells (Figure 1-ii.b).

### *L. rhamnosus* could co-aggregate with *P. gingivalis*

The results showed that the strains exhibited a strong auto-aggregation phenotype (Table 1). The co-aggregation of *L. rhamnosus* with *P. gingivalis* is expressed as the percentage reduction after 4 h in the absorbance of a mixed suspension, compared with the individual suspension.

**Table 1:** Auto-aggregation and co-aggregation ability of strains

	Auto-aggregation (%)		Co-aggregation (%)
Strains	*L. rhamnosus*	*P. gingivalis*	*L. rhamnosus* and *P. gingivalis*
	98.8±0.0001**	95.25±0.002	23.7±0.002

*A negative significant correlation was found between *L. rhamnosus* and *P. gingivalis* co-aggregation according to Spearman's rho test

**± refers to the standard deviation

### *L. rhamnosus* inhibits the adhesion of *P. gingivalis*

The morphology of G-MSSCs following treatment with a viable probiotic strain and *P. gingivalis* was examined by light microscopy. No obvious morphological changes induced by the bacteria were observed. We distinguished the attached *P. gingivalis* from the probiotic strain according to their Gram stain properties. We also determined the adhesion of the strains separately; *L. rhamnosus* showed higher adhesive properties than *P. gingivalis* on G-MSSCs (p<0.01; Table 2).

**Table 2:** Adhesion ability of strains

	Adhesion ability (bacteria/cell)*	Competitive adhesion ability (bacteria/cell)**
L. rhamnosus*	34.02±0.25***	22±2
*P. gingivalis*	6.85±0.1	2±0.2

*Difference between *L. rhamnosus* and *P. gingivalis* adhesion on G-MSSCs was found to be highly significant according to Mann-Whitney U test (p<0.01 and p<0.05)

**Competitive adhesion was not found to be statistically significant according to Mann-Whitney U test (p>0.01 and p>0.05)

***± refers to the standard deviation

### *L. rhamnosus* strains could manage to induce CXCL8

**Figure 2:**
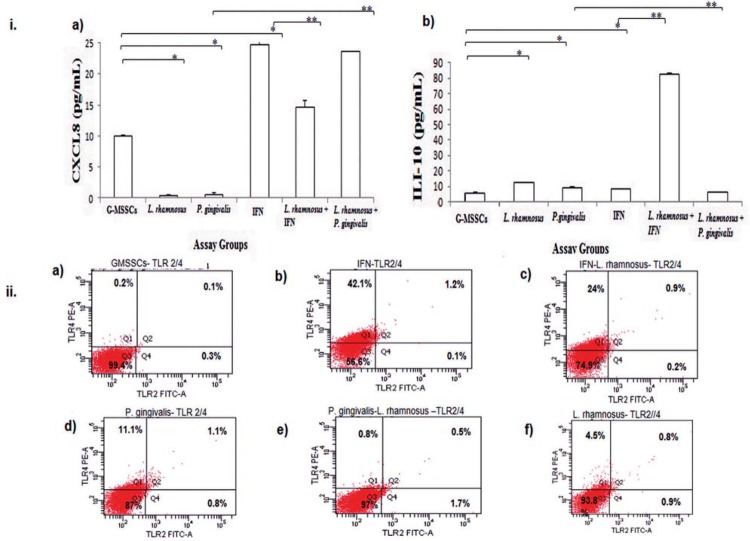
i. *L. rhamnosus* ATCC9595 modulated CXCL8 (a) and IL-10 (b) secretion. (a) The gingival stromal stem cells (G-MSSCs) secreted an amount of CXCL8 without any stimulation. *L. rhamnosus* and *P. gingivalis* reduced CXCL8, as known. The reduction was found to be significant according to Mann-Whitney U test (p<0.05). A *L. rhamnosus* and *P. gingivalis* coculture exhibited a stimulant effect for CXCL8 as an inflammatory inducer, according to a Kruskal-Wallis test, which verified our hypothesis (p<0.05). IFN provoked GMSSCs to secrete CXCL8 (p<0.05). On the other hand, CXCL8 was found to be reduced on *L. rhamnosus* pretreated G-MSSCs when induced with IFN (p<0.05). (b) In contrast to CXCL8, the G-MSSCs did not secrete IL-10. *L. rhamnosus* and *P. gingivalis* increased IL-10 secretion, compared with G-MSSCs (p<0.05). The *L. rhamnosus* and *P. gingivalis* coculture reduced IL-10, since CXCL8 was increased (p<0.05). IL-10 was reduced on IFN-induced G-MSSCs, while CXCL8 was increased. *L. rhamnosus* -pretreated G-MSSCs induced IL-10 in IFN stimulation (p<0.05). ii . TLR expression was found to be synchronized with CXCL8 and IL-10 secretion. (a) G-MSSCs did not express TLR2 or 4 (<99.3%) b) G-MSSCs expressed TLR4 (42.1%) when stimulated with IFN (p<0.01). c) G-MSSCs pretreated with *L. rhamnosus* decreased the expression of TLR4 (24%) in IFN-induced inflammatory conditions (p<0.01). d) *P. gingivalis* -induced TLR4 expression (11.1%; p<0.01). On the other hand, both TLR4 and TLR2 were expressed (1.1%). Decreased CXCL8 represents a gingipain effect, since we expected increased CXCL8 due to expressed TLR4 e) The *L. rhamnosus* and *P. gingivalis* coculture was able to reduce TLR4 expression to 0.5% (p<0.01). On the other hand, TLR2 was found to be 1.7% (p<0.01), which indicates the TLR2- dependent CXCL8 secretion. f) *L. rhamnosus*, when used alone, could induce TLR4 (4.5%) (p<0.01). G-MSSCs: Gingival mesenchymal stromal stem cell. IFN: Interpheron –γ. * indicates statistically significant groups

Both CXCL8 (Figure 2-i.a) and IL-10 (Figure 2-i.b) were found in the G-MSSC cell culture supernatants. Our findings showed that when used alone, *L. rhamnosus* and *P. gingivalis* reduced CXCL8 levels and increased IL-10 in cell culture (p<0.05). As expected, IFN-γ increased CXCL8 (21 pg mL^-1^) and decreased IL-10 (4.4 pg mL^-1^) levels in G-MSSCs (p<0.05). The probiotic strain was able to control the CXCL8 level against IFN-γ stimulation (reduced to 14 pg mL^-1^) and induced IL-10 levels (9.26 pg mL^-1^; p<0.05). On the other hand, when G-MSSCs were pretreated with *L. rhamnosus* before *P. gingivalis* stimulation, CXCL8 secretions were found to increase to 22 pg mL-1 (p<0.05).

### *L. rhamnosus* reduces TLR-4 expression on exposure to IFN-γ and *P. gingivalis*

We then examined the TLR-2 and TLR-4 expression of the G-MSSCs using flow cytometry (Figure 2-ii). The G-MSSCs upregulated the expression of TLR-4 on IFN-γ and *P. gingivalis* stimulation (Figure 2-ii.d; 42.1% and 11.1%, respectively), compared with the unstimulated group (Figure 4a; p<0.01). *P. gingivalis* also induced as much TLR-2 expression as the probiotic strain (0.8%; p<0.01). *L. rhamnosus* reduced *P. gingivalis*-induced TLR-4 expression from 11.1% to 0.8% (p<0.01; Figure 2-ii.e). Also, the G-MSSCs pretreated with *L. rhamnosus* downregulated TLR-4 expression to 24% against IFN-γ stimulation (p<0.01) (Figure 2-ii.c). *P. gingivalis* induced both TLR-4 and TLR-2 expression in 1.1% of the G-MSSCs (Figure 2-ii.d). This value was reduced to 0.5% when the probiotic strain was pretreated in this group. An analogous result was seen in IFN-γ-stimulated cells (1.2%) (Figure 2-ii.b). The probiotic strain was able to reduce the number of cells expressing both TLR-4 and TLR-2 (0.9%; Figure 2-ii.f). On the other hand, when used alone, the probiotic strain caused 4.5% of the GMSSCs to express TLR-4 and 0.8% of the cells to express TLR-2.

## DISCUSSION

This *in vitro* study aimed to investigate whether the probiotic strain *L. rhamnosus* ATCC 9595 could prevent the *P. gingivalis* welded CXCL8 supression through co-aggregation, competitive adhesion, and the expression of TLRs. We chose to use the probiotic *L. rhamnosus* because the strain was reported to induce IL-10 secretion and produce high levels of exopolysaccharides (EPSs) in different works^4^ ^,^ ^6^. EPSs are one of the primary metabolic products of lactic acid bacteria, and have received an increasing amount of attention because of their health benefits^22^. The ability of a microorganism to surround itself with a highly hydrated EPS layer may provide it with protection against desiccation and predation. We suggest that in terms of oral environments in clinical studies, considering the stressful conditions created by saliva and tooth surfaces, high EPS-productive strains of *L. rhamnosus* may survive better.

Although the mechanisms of action are not fully understood, it is generally accepted that the ability of probiotics to co-aggregate with pathogens is a desired property. Co-aggregation has an important ecological role as an integral process in the development and maintenance of mixed-species biofilm communities, especially in oral cavity. In order to exhibit beneficial effects, probiotic bacteria need to achieve an adequate mass through aggregation. We used a simple and robust spectrophotometric method that has been shown to correspond well to more sophisticated radioactive labeling techniques. The findings indicated that *L. rhamnosus* could co-aggregate with *P. gingivalis* after 4 h. The co-aggregation ability of the probiotic strain could enable the formation of a barrier that prevents colonization by pathogenic bacteria^9^.

In addition to aggregation, attachment is an important function for bacteria in forming biofilms, and in most cases, aggregation ability is related to cell adherence properties^9^. We examined the competitive adhesion of the probiotic strain and *P. gingivalis* on G-MSSC monolayers that were grown on glass coverslips. To our knowledge this is the first study investigating *L. rhamnosus* and *P. gingivalis’*s competitive adhesion on G-MSSCs. *L. rhamnosus* exhibited the strongest affinity with G-MSSCs, which is in good agreement with studies conducted with Caco-2 cells^11^ ^,^ ^27^. It is feasible to take advantage of *L. rhamnosus*, which could confer advantage to this strain a competitive *in vivo*. It is well known that *P. gingivalis* adheres to and invades epithelial cells or fibroblasts *in vitro*. The invasion of epithelial cells, as well as gingival fibroblasts, is probably a mechanism applied by bacteria to evade the immune system of the host^1^. It was reported that invasion could occur after 6 h of incubation^25^. Based on these reports, we incubated the probiotic strain and *P. gingivalis* for 1 h to prevent the invasion into the GMSSCs*.* We showed that the probiotic strain inhibited the adhesion of *P. gingivalis*, although the mechanism remains unclear. Our results do not explain whether the exclusion of *P. gingivalis* was due to a competition for specific sites on the surface of G-MSSCs or the constitution of a biofilm of bacteria prevented access to the cell surfaces of organisms. These results show a potential for adhesive and co-aggregative *L. rhamnosus* to inhibit the cell association and cell entry of *P. gingivalis.* Many studies have tried to induce a microbiological shift or a clinical probiotic effect in an already matured oral microbiological environment. However, it seems logical that a probiotic will have difficulty in colonizing the mouth and exerting beneficial clinical effects. We suggest that *L. rhamnosus* with its co-aggregation ability and strong adhesive properties, may survive better.

As a consequence of adhesion, the preference would orient the cell response according to the probiotic strain, since probiotics can also activate and modulate the immune system^7^. In the present study, we demonstrated that probiotic *P. gingivalis* interactions can inhibit CXCL8 attenuation. It is known that secreted CXCL8 proteins are downregulated when cells are challenged with *P. gingivalis* ^14^. The suggested mechanism comprises the downregulation of CXCL8-mRNA and/or the degradation of CXCL8 by proteases (called gingipains). Strong evidence has shown that using antibiotics or deleting one of the protease genes (rgpA, rgpB, or kgp) in *P. gingivalis* did not dramatically affect CXCL8 attenuation^14^. We showed that 12 h of exposure to viable *P. gingivalis* suppressed CXCL8 production in the cells, which is inconsistent with literature^17^ ^,^ ^25^. On the other hand, when the cells were pretreated with the probiotic strain before *P. gingivalis* stimulation, the CXCL8 levels were found to have increased, indicating that *P. gingivalis* proteases might be degraded by *L. rhamnosus* or *P. gingivalis*-probiotic strain co-aggregation, which may activate or deactivate any structures on the bacteria cell wall responsible for the degradation of CXCL8. Indeed, we need to demonstrate the molecular mechanism in this interaction. We screened the effects of bacteria on cytokine secretions when used alone. Low levels of CXCL8 and IL-10 were determined in the G-MSSCs culture supernatant, as corroborated by Tonetti, et al.^28^ (1994), who reported that low-level expression of CXCL8 in healthy tissue most likely contributes to the remarkable ability of the host to limit periodontal bacterial growth. To further investigate the effect of probiotics on CXCL8 secretion, G-MSSCs were stimulated with IFN-γ, a well-known inducer of inflammatory mediators. Conversely, during IFN-γ stimulation, CXCL8 levels were downregulated and IL-10 levels were found to have increased. Such immunomodulation induced by probiotics is important for maintaining the host-microbe homeostasis without triggering exaggerated, detrimental inflammatory responses.

Because TLR activation plays a vital role in cytokine production, we measured the expression of TLRs after treatment with the probiotic strain and *P. gingivalis*. It must be noted that *P. gingivalis* lipopolysaccharide (LPS), another putative virulence factor, is suggested to evade recognition by the host via TLR-4^29^. The lack of significant TLR-4 involvement in the host response to *P. gingivalis* is counterintuitive, since that this is a gram negative bacterium that expresses a LPS. However, *P gingivalis* utilizes a specific lipid 1- and 4-phosphatases and a deacylase, which, in concert, generate a tetracylated and dephosphorylated lipid, a structure that is biologically inert^7^. In contrast to this report, in our study we observed TLR-4 expression (11.1%), but reduced CXCL8 levels and TLR-2. This result could be explained by the degradation of secreted CXCL8 by gingipains. Moreover, *P. gingivalis*’s effect was clearly altered when *L. rhamnosus* was added to the culture. CXCL8 and TLR-2 were found to be enhanced, while TLR-4 expression was reduced. Several groups have found that TLR-2 is required for a full cytokine response to infection with *P. gingivalis*, raising the question of whether *P. gingivalis* evades clearance by reducing recognition through TLR-2, rather than TLR-4^5^ ^,^ ^13^. Our results clearly showed that *L. rhamnosus* together with *P. gingivalis* boosts CXCL8 production while enhancing TLR 2 and inhibiting TLR-4 expression. Previous reports showed TLR-2, rather than TLR-4, to be critical for the host response to infection with *P. gingivalis* ^5^ ^,^ ^13^. Gingipain inhibition by *L. rhamnosus* and its effect on TLR-2 expression should be determined to conclusively show that *L. rhamnosus* inhibits CXCL8 attenuation through an enzyme or polysaccharides.

## CONCLUSION

The immunomodulatory probiotic strain *L. rhamnosus* ATCC 9595 is proposed to be essential for maintaining healthy tissue, with multiple roles including co-aggregation, adhesion, and priming immune responses to ensure rapid and efficient defense against *P. gingivalis* ATCC 33277. The default state of oral tissues, such as in the gut, is an inflammation that may be balanced by regulatory mechanisms and the activities of anti-inflammatory probiotics that modulate TLR signaling. There are significant discrepancies among studies that evaluated the effects of probiotics, possibly because they used different doses, treatment durations, bacterial species, and application forms. These conflicting results point out that not all probiotics have beneficial effects on periodontal diseases. Therefore, it seems necessary to perform specific screenings to select appropriate probiotic strains for preventing gingivitis or periodontitis and other oral health diseases. Thus, new *in vitro* studies with greater scientific rigor should be developed to evaluate the safety and efficacy of probiotics before they are introduced into clinical practice for periodontal therapy. The results of the present work provide a scientific rationale for the use of *L. rhamnosus* ATCC 9595 to prevent *P. gingivalis* induced inflammation and CXCL8 attenuation. However, our results should be proven *in vivo*, through both human and animal trials.
